# Effect of high-fructose consumption in pregnancy on the bone growth of offspring rats

**DOI:** 10.3389/fnut.2023.1203063

**Published:** 2023-08-17

**Authors:** Yijing Li, Xiaoqian Liu, Yuning Chu, Cai Li, Tianlin Gao, Xiuli Jiang, Zihan Zhu, Qi Sheng, Lei Han

**Affiliations:** ^1^Department of Nutrition, The Affiliated Hospital of Qingdao University, Qingdao, China; ^2^Maternal, Child & Adolescent Health, Qingdao University, Qingdao, China; ^3^School of Public Health, Qingdao University, Qingdao, China

**Keywords:** fructose, gestation, offspring, bone development, micro-CT

## Abstract

Growing evidence suggests that bone health is programmed in early life. Maternal diet may influence the skeletal development of offspring. We aimed to determine the possible effects of high-fructose intake during pregnancy on different aspects of long bone morphology in the offspring of rats and to initially explore the possible mechanisms. Pregnant Sprague-Dawley rats were randomly divided into four groups and intragastrically administered the same dose of distilled water (CON, *n* = 12), 20 g/kg/day glucose (GLU, *n* = 12), 10 g/kg/day fructose (LFRU, *n* = 12), or 20 g/kg/day fructose (HFRU, *n* = 12) for 21 days during gestation. Computed tomography was used to analyze the cortical and cancellous bones of the distal femur of the offspring rats, and circulating bone metabolic biomarkers were measured using enzyme immunoassay. The results showed that high-fructose intake during pregnancy could decrease body weight, impair glucose metabolism, and increase serum leptin and uric acid in offspring. The offspring in the HFRU group had higher levels of the N-terminal propeptide of type I procollagen (PINP) and the C-telopeptide of type I collagen (CTX). The bone mean density (BMD), the total cross-sectional area inside the periosteal envelope (Tt.Ar), cortical bone area (Ct.Ar), medullary (or marrow) area (Ma.Ar), and trabecular mean density of the offspring in the HFRU group were lower than those in the CON group. Tartrate-resistant acid phosphatase (Trap) staining showed that high-fructose intake during pregnancy could increase the number of osteoclasts and increase the absorption area. Our results suggested that excessive fructose intake during pregnancy could inhibit skeletal development in offspring. Thus, attention to fructose intake during pregnancy is important for bone development in offspring.

## 1. Introduction

Fructose is an isomer of glucose that exists in fruit and honey. Different from glucose, fructose does not stimulate insulin release. Following lactate production and glycogen synthesis after ingestion, fructose causes intracellular ATP depletion, nucleotide turnover, and uric acid production (1). Due to the excess intake of fruit, as well as processed foods and beverages that use fructose as a sweetener, dietary fructose intake has increased significantly (2). The production of high fructose corn syrup (HFCS) in China has grown rapidly over the past decade, from 1.4 million tons in 2011/2012 to 3.2 million tons in 2018/2019, an increase of 128.6% (3). Studies have shown that the highest fruit intake of pregnant women in one region of China was 390–699 g, and the rates of beverage intake among pregnant women in early and mid-pregnancy were 13 and 8.9%, respectively (4, 5).

The effect of fructose on health has attracted increasing attention. Fructose intake can also affect individual bone development. Our previous study showed that a group administered 20% fructose had a lower bone volumetric fraction (BV/TV) and trabecular thickness (Tb.Th) than the control group, which suggested that excessive fructose intake may inhibit bone growth and reduce trabecular bone density (6). Other research has indicated that 30% HFCS in water could cause damage to the trabecular bone with decreases in the trabecular number (Tb.N) and the trabecular thickness (Tb.Th), as well as an increase in the bone volumetric fraction (Tb.Sp) (7). Although a high fructose diet (35% energy from fructose) has been reported to first increase and then decrease bone mass (8), there are few reports on the effect of high fructose consumption during pregnancy on offspring bone.

In humans, the skeleton begins to develop early in pregnancy. The fetal skeleton grows rapidly in the uterus and continues to grow rapidly after birth before slowing in infancy (9). The Developmental Origins of Health and Disease (DOHaD) hypothesis states that a variety of adverse factors experienced during early development can contribute to the development of multiple adult-period chronic non-infectious diseases (NCDs), including osteoporosis (10). It has been suggested that the nutritional status of pregnant women directly or indirectly affects the growth of fetal bone, although the mechanism is still unclear (11). For example, high-fat-mediated obesity in mice during gestation may decrease fetal bone metabolism by enhancing the expression of fetal osteoblastic cell senescence signals (12).

Excessive maternal fructose intake can negatively affect embryonic development and offspring health and influence fetal development by altering the intrauterine environment and placental transport, leading to low birth weight and even adverse pregnancy outcomes (13). Maternal intake of a high-fructose diet during pregnancy alters the oxidative stress and metabolic status of the offspring (14–16), which are important factors in skeletal growth and development. Further exploration of the effects of excessive fructose intake during pregnancy on the mother and offspring and the specific mechanisms leading to these adverse outcomes is needed.

Therefore, we conducted animal studies to explore the effects of maternal high-fructose intake during pregnancy on bone formation, morphology, and quality in offspring. We monitored biomarkers of the bone metabolic microenvironment and bone histomorphometry parameters, including bone mass and bone microstructure, in the offspring of rats with high maternal fructose intake during pregnancy.

## 2. Materials and methods

### 2.1. Animals and experimental design

A total of 48 female Sprague-Dawley rats (210 ± 10 g, Charles River) were housed in a temperature- and humidity-controlled room with a 12-h light/dark cycle. The female rats were mated overnight with male rats. Successful mating was defined as the day when the presence of a sperm-positive vaginal smear was found, which was designated as embryonic day 0 (ED0). Matched for body weight, the pregnant rats were randomly assigned to the CON group (*n* = 12), GLU group (*n* = 12), LFRU group (*n* = 12), and HFRU group (*n* = 12). The CON group was provided with water with no supplementary sugar by gavage. The GLU group intragastrically received 20 g/kg glucose solution two times a day throughout the pregnancy. The LFRU and HFRU groups intragastrically received 10 g/kg or 20 g/kg of fructose solution two times a day throughout the pregnancy. All pregnant rats were fed standard rodent chow (AIN-93G, Keaoxieli Fodder, Beijing, China) and were housed individually. Food and water with no supplementary sugar were freely available. Food intake was measured three times a week. Body weights were measured weekly. On ED21, half of the dams were weighed and fasted overnight. Oral glucose tolerance tests (OGTTs) were performed at 8:00 a.m., the next morning in dams. Fasting blood glucose (FBG) was measured first. After intragastric administration of a 50% glucose solution at 2.0 g/kg body weight, blood glucose was measured at 30 min, 60 min, and 120 min. Blood was collected, and serum was separated and stored at −80°C. Samples were collected from the liver, gastrocnemius, and femur. The daily energy intake (DEI) per pregnant rat (kcal/day/rat) was calculated as follows: daily feed intake × 3.85 kcal/g + fructose dose × 4 kcal/g.

The rest of the dams giving birth naturally were provided with water with no supplementary sugar during lactation. Food intake was measured twice a week. To standardize the feeding protocol, each litter was adjusted to 8 pups (4 female and 4 male pups). The pups were fed until weaned at 3 weeks of age. Finally, 16 offspring rats (8 male and 8 female pups) were randomly selected from each group to continue feeding for 4 weeks and for research. Offspring were fed a control diet (AIN-93G, Keaoxieli Fodder, Beijing, China) and water with no supplementary sugar at 3 weeks of age and followed until 7 weeks of age. The offspring's food intake was measured three times a week. The offspring's body weight was measured weekly. Oral glucose tolerance tests (OGTTs) were performed at 8:00 a.m. the next morning in offspring at 7 weeks of age. Offspring at 7 weeks of age were then anesthetized. Blood was collected, and serum was separated and stored at −80°C. Samples were collected from the liver, gastrocnemius, femur, and tibia. The daily energy intake (DEI) per lactating rat and offspring rat (kcal/day/rat) was calculated as daily feed intake × 3.85 kcal/g. All experimental procedures and protocols followed the guidelines for the care and use of animals established at the Medical College of Qingdao University and approved by the Animal Experimentation Ethics Committee of the Medical College of Qingdao University.

### 2.2. Serum measurements

ELISA kits were used to determine the concentrations of fasting insulin (FIN), fructose, uric acid (UA) (from Jiancheng Technology, Nanjing, China), leptin, N-terminal propeptide of type I procollagen (PINP), and C-telopeptide of type I collagen (CTX) (from Jingkang Technology, Shanghai, China). The homeostasis model assessment of insulin resistance (HOMA-IR) value was calculated as FBG (mmol/L) × FIN (mU/L)]/22.5. The area under the curve (AUC) and the incremental AUC were used to estimate the total glucose increase during the OGTT.

### 2.3. Microcomputed tomography

#### 2.3.1. Scanning, reconstruction, and image processing

The distal region of the femur was scanned on a desktop X-ray microtomograph (Quantum GX micro-CT Imaging) at Qingdao University facilities. The femora were imaged with an X-ray tube voltage of 90 kV and a current of 80 μA, with an acquisition FOV of 36 μm and a high-resolution scan mode for 14 min. Datasets were reconstructed using the Quantum GX imaging system. For the analysis of microstructural parameters of cancellous and cortical bone, the region of interest (ROI) started at 200 consecutive sections from the growth plate of the distal femur (taken as the reference section) and included 120 consecutive sections in the proximal direction. The selection of the corresponding ROIs and consequent bone microstructural properties and vBMD analyses were carried out with the commercial software provided with the micro-CT equipment (Analyze 12.0). Bone measurements were obtained by staff who were blinded to the treatment group of the rats.

#### 2.3.2. Volumetric bone mineral density

In both ROIs, BMD (g/cm^3^) was determined using micro-CT by direct comparison with the attenuation coefficients of five hydroxyapatite phantoms with known densities (1.13, 1.17, 1.27, 1.65, and 1.91 g/cm^3^) used as patterns.

#### 2.3.3. Microstructural parameters of cortical bone

Cortical bone parameters were measured using micro-CT from the values obtained by analyzing each individual transverse section (2D analysis) and accounting for the space between them (3D analysis). Parameters obtained by 3D analysis included the cortical porosity Po.V/CT.V (Ct.Po, %), pore number (Po.N, #), total pore volume (Po.V, mm^3^), and pore density Po.N/CT.V (Po.Dn, mm^−3^). Parameters obtained by 2D analysis included total cross-sectional area inside the periosteal envelope (Tt.Ar, mm^2^), cortical bone area (Ct.Ar, mm^2^), medullary (or marrow) area (Ma.Ar, mm^2^), cortical area fraction (Ct.Ar/Tt.Ar, %), average cortical thickness (Ct.th, mm), periosteal perimeter (Ps.Pm, mm), endocortical perimeter (Ec.Pm, mm), and polar moment inertia (Jo, mm^4^).

#### 2.3.4. Microstructural parameters of cancellous bone

Morphometric indices of the cancellous bone region were determined using the datasets integrated over an ROI. The bone volumetric fraction (BV/TV %), bone surface density (BS/TV, mm^−2^), and specific bone surface (BS/BV, mm^−2^) were calculated directly. BS/TV is the ratio of surface area to total volume measured, whereas BS/BV is the ratio of the bone surface per given bone volume, which provides a measure of how many bone lining cells cover a given volume of bone. Parameters obtained by 3D analysis included trabecular thickness (Tb.Th, mm), trabecular spacing (Tb.Sp, mm), and connectivity density (Conn.D, mm^−3^). Parameters obtained by 2D analysis included trabecular number (Tb.N, mm^−1^), degree of anisotropy (DA, %), and mean intercept length (MIL, %).

### 2.4. Femur staining

Femur tissues were fixed in 10% formalin phosphate at the time of collection, decalcified, and embedded in paraffin. The femurs were sliced, soaked in hematoxylin, eosin solution, and Tartrate resistant acid phosphatase (Trap) solution respectively (Servicebio Technology, Wuhan, China), and then rinsed and drained. The slices were dehydrated with anhydrous ethanol and sealed with neutral gum. Trap staining was used to observe the osteoclasts in the femurs of rats, and staining showed that the red overlying layer was osteoclasts. The stained sections were observed using an optical microscope (BX53, Olympus, Japan) with a 10 × eyepiece and 40 × objective lens. Three femurs from different groups were selected for analysis in each group. ImageJ software (version 1.53 K, USA) was used for the analyses of the Trap cell area.

### 2.5. Statistical analysis

SPSS (version 26.0) was used for statistical analysis of the data, and *P* < 0.05 was considered statistically significant. The distribution of all data was assessed using the Kolmogorov–Smirnov test for normality. The data satisfying the normal distribution were described in the form of mean ± standard deviation, and the homogeneity test of variance was conducted. If the data satisfied the test for homogeneity of variance, one-way analysis of variance (ANOVA) was used for multiple groups, and the Bonferroni method was used for between-group (pairwise) comparisons. If the data did not satisfy the test for homogeneity of variance, the Welch test was used for multiple-group comparisons, and the Tamhane method was used for between-group comparisons. The GraphPad Prism (version 8.0) was used for graphing and analysis, and data with differences are marked with symbols.

## 3. Results

### 3.1. Body weight and food intake of dams

**Figure 1:**
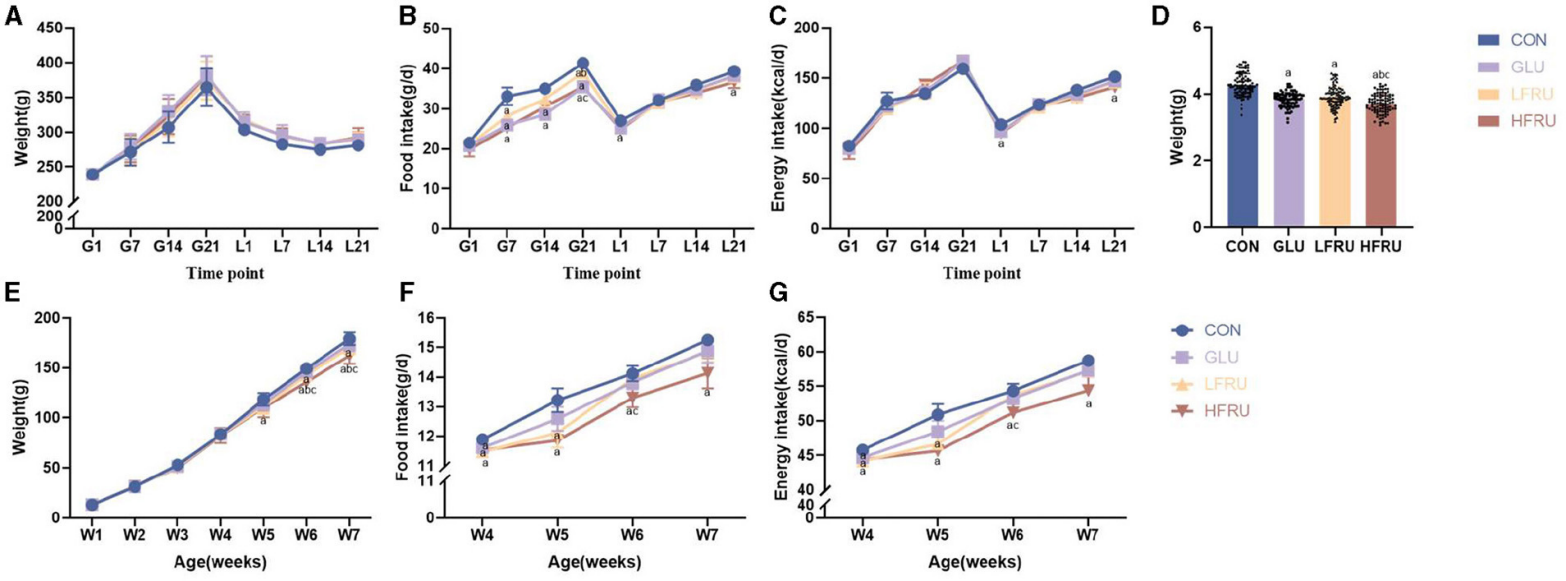
Dams and offspring weight, food intake, and energy intake. **(A)** Dam body weight during gestation and lactation. **(B)** Dam food intake during gestation and lactation. **(C)** Dam energy intake during gestation and lactation. **(D)** Offspring body weight at gestational day 21. **(E)** Offspring weight gain for 7 weeks. **(F)** Offspring food intake after parturition. **(G)** Offspring energy intake after parturition. Values are the means ± SDs, *n* = 12 dams per group during gestation, *n* = 6 dams per group during lactation, *n* = 8 female and 8 male offspring per group. G, gestation; L, lactation; W, week. ^a^*P* < 0.05 (HFRU, LFRU, and GLU vs. CON). ^b^*P* < 0.05 (HFRU and LFRU vs. GLU). ^c^*P* < 0.05 (HFRU vs. LFRU).

The body weight, food intake, and energy intake of dams during gestation and lactation are shown in Figure 1. There was no difference in body weight, liver weight, gastrocnemius weight, or femur weight between dams (Figure 1A, Table 1). In addition, we observed significantly lower food intake during gestation in the GLU, LFRU, and HFRU groups than in the CON group, indicating that the animals consumed less food when receiving glucose and fructose (Figure 1B). However, considering the consumption of glucose and fructose, no significant difference in energy intake was observed during gestation (Figure 1C). During 21 days of lactation, the food intake and energy intake of dams increased with increasing time (Figures 1B, C). Compared with the CON group, food intake and energy intake were significantly reduced in the HFRU group on days 1 and 21 of lactation (Figures 1B, C).

**Table 1:** Anthropometrics of dams at parturition.

**Treatment**	**CON**	**GLU**	**LFRU**	**HFRU**
Liver weight (g)	11.35 ± 1.57	11.14 ± 0.74	11.07 ± 1.23	11.96 ± 1.38
Gastrocnemius weight (g)	0.13 ± 0.01	0.13 ± 0.01	0.13 ± 0.01	0.13 ± 0.02
Femur weight (g)	1.18 ± 0.24	1.14 ± 0.17	1.17 ± 0.22	1.22 ± 0.16

### 3.2. Body weight and food intake of offspring in early life

Compared with the CON group, the GLU, LFRU, and HFRU groups had significantly lower pup birth weights at 21 days of pregnancy (Figure 1D). Offspring rat body weights increased with prolonged feeding time (Figure 1E). The HFRU group gained weight at weeks 6 and 7 much less than the CON and GLU groups (Figure 1E). In addition, the HFRU group showed considerably decreased food consumption and energy intake (Figures 1F, G). Meanwhile, the femur bone weight of offspring male rats in the HFRU group was significantly lower than that in the GLU group, the gastrocnemius muscle weight and the femur bone weight of offspring female rats in the HFRU group were significantly lower than that in the CON group, and the tibial bone weight of offspring female and male rats in the HFRU, LFRU, and GLU groups was significantly lower than that in the CON group (Table 2). There was no significant difference in the weight of the liver among the offspring male and female groups or in the weight of the gastrocnemius among the offspring male groups.

**Table 2:** Anthropometrics of offspring stratified by sex.

	**Treatment**	**CON**	**GLU**	**LFRU**	**HFRU**
	Liver weight (g)	6.38 ± 1.04	6.35 ± 1.00	6.36 ± 1.14	6.06 ± 1.04
Male	Gastrocnemius weight (g)	1.04 ± 0.14	1.03 ± 0.10	1.01 ± 0.15	0.88 ± 0.14
	Femur weight (g)	0.61 ± 0.06	0.64 ± 0.16	0.55 ± 0.11	0.47 ± 0.09^b^
	Tibial weight (g)	0.49 ± 0.08	0.36 ± 0.08^a^	0.33 ± 0.05^a^	0.28 ± 0.05^a^
	Liver weight (g)	6.16 ± 0.89	6.19 ± 0.98	6.16 ± 1.21	6.00 ± 0.92
Female	Gastrocnemius weight (g)	1.04 ± 0.06	1.00 ± 0.12	0.96 ± 0.15	0.85 ± 0.11^a^
	Femur weight (g)	0.62 ± 0.09	0.55 ± 0.09	0.52 ± 0.13	0.43 ± 0.10^a^
	Tibial weight (g)	0.42 ± 0.07	0.32 ± 0.04^a^	0.32 ± 0.06^a^	0.30 ± 0.08^a^

Values are the means ± SDs, *n* = 8 female and 8 male offspring per group. ^a^*P* < 0.05 (HFRU, LFRU, and GLU vs. CON). ^b^*P* < 0.05 (HFRU and LFRU vs. GLU).

### 3.3. Peripheral glucose regulation in dams and offspring

**Figure 2:**
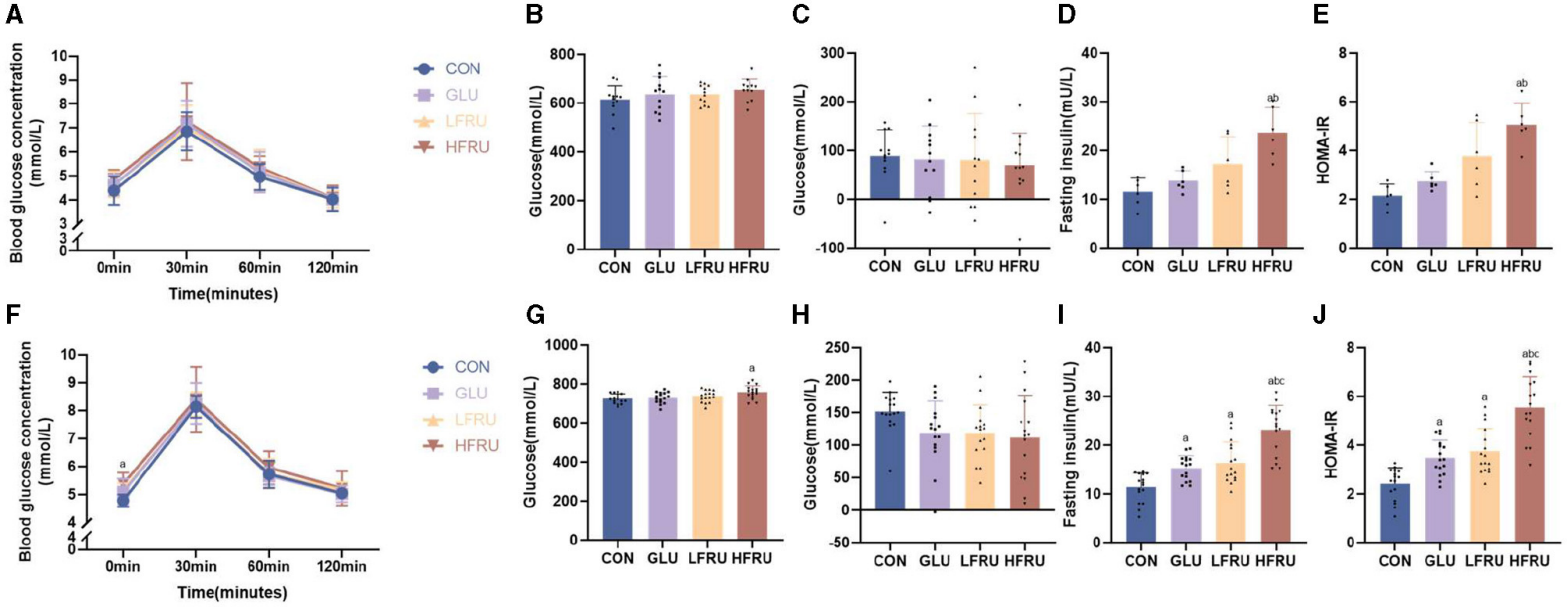
Glucose tolerance, fasting insulin concentrations, and HOMA-IR of dams and offspring. **(A)** Dam glucose response during the OGTT. **(B)** AUC of the OGTT in dams. **(C)** Incremental AUC of the OGTT in dams. **(D)** Dam fasting insulin concentrations. **(E)** HOMA-IR levels in dams. **(F)** Offspring glucose response during the OGTT. **(G)** AUC of the OGTT in offspring. **(H)** Incremental AUC of the OGTT in offspring. **(I)** Offspring fasting insulin concentrations. **(J)** HOMA-IR levels of offspring. Values are the means ± SDs. *n* = 12 dams per group during gestation, *n* = 8 female and 8 male offspring per group. OGTT, oral glucose tolerance test. ^a^*P* < 0.05 (HFRU, LFRU, and GLU vs. CON). ^b^*P* < 0.05 (HFRU and LFRU vs. GLU). ^c^*P* < 0.05 (HFRU vs. LFRU).

Glycemia during OGTT in dams at gestation was not affected by fructose intake (Figure 2A). There was no significant difference in the glucose AUC and the incremental AUC in dams among the groups (Figures 2B, C). These data indicated that fructose supplementation for 3 weeks in dams did not affect glucose homeostasis in dams. However, compared with the CON and GLU groups, the fasting serum insulin concentration and HOMA-IR of pregnant rats in the HFRU group were significantly increased (Figures 2D, E).

The OGTT results of offspring rats among the four groups showed that although there was no significant difference in serum blood glucose concentration at 30 min, 60 min, and 120 min of OGTT, the fasting blood glucose concentration of offspring rats in the HFRU group (HFRU = “5.38 ± 0.41” vs. CON = “4.78 ± 0.22” cont) and LFRU group (LFRU = “5.16 ± 0.24” vs. CON = “4.78 ± 0.22” cont) increased significantly compared with the CON group (Figure 2F). The HFRU group of offspring also showed a substantially greater glucose AUC compared to the CON group (Figure 2G). However, there was no difference in the incremental AUC among the four groups (Figure 2H). Comparing the HFRU group to the CON, GLU, and LFRU groups, we found that the HFRU group of offspring had significantly higher serum insulin concentrations and HOMA-IR levels (Figures 2I, J), indicating that fructose supplementation for 3 weeks in dams may have an impact on the offspring's insulin sensitivity at 7 weeks of age.

### 3.4. Fructose metabolism in dams and offspring

**Figure 3:**
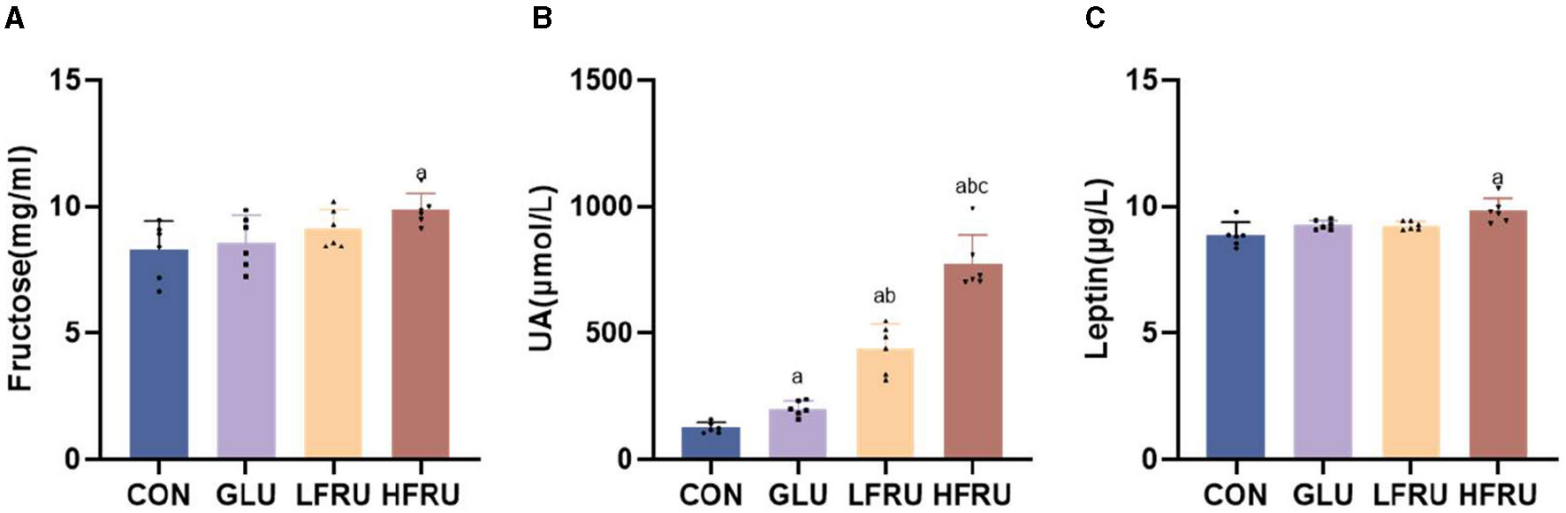
Serum fructose, UA, and leptin concentrations of dams. **(A)** Serum fructose concentrations. **(B)** UA concentrations. **(C)** Leptin concentrations. Values are the means ± SDs. *n* = 6 dams per group, ^a^*P* < 0.05 (HFRU, LFRU, and GLU vs. CON). ^b^*P* < 0.05 (HFRU and LFRU vs. GLU). ^c^*P* < 0.05 (HFRU vs. LFRU).

The serum fructose concentration of pregnant rats in the HFRU group was significantly increased compared with the CON group (Figure 3A). Fructose supplementation resulted in a significant increase in UA in dams in the HFRU, LFRU, and GLU groups (Figure 3B).

**Figure 4:**
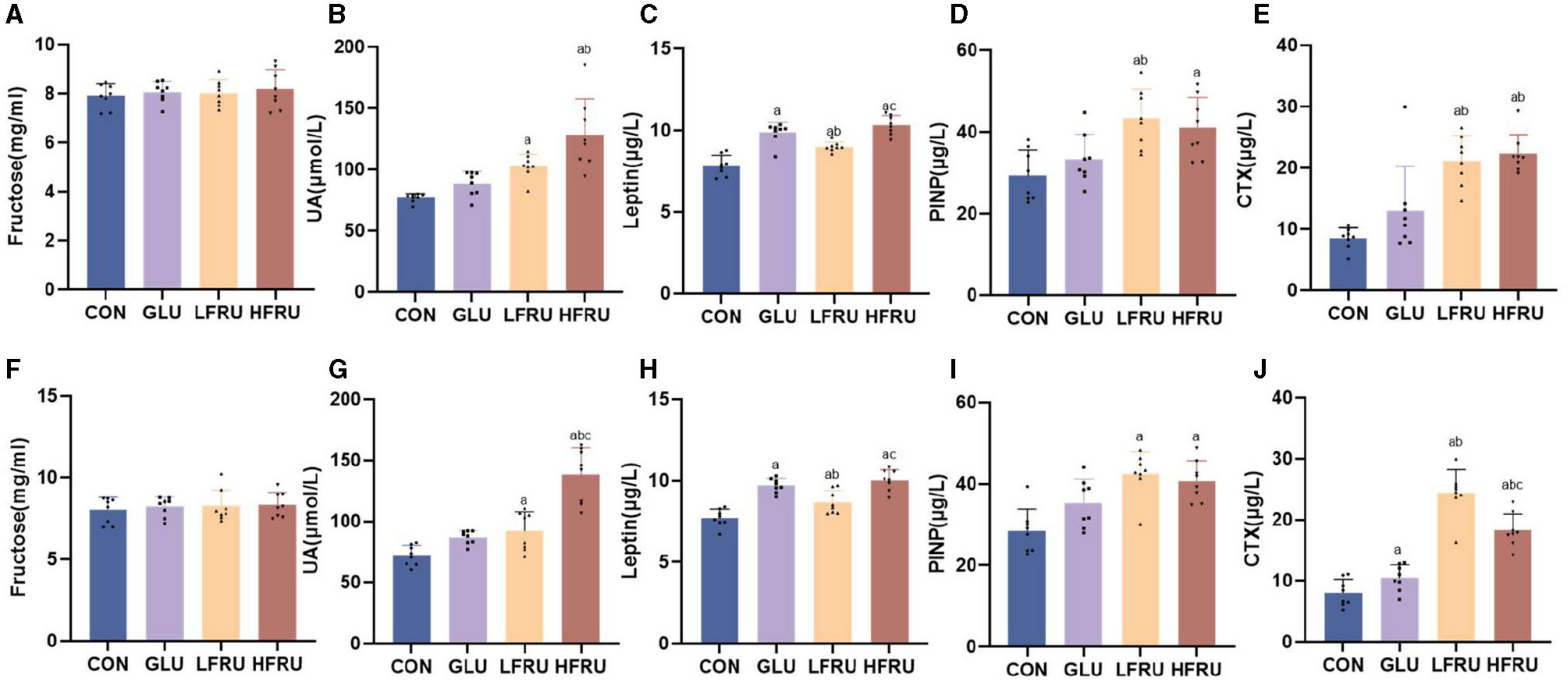
Serum fructose, UA, leptin concentrations, and circulating markers of bone metabolism of offspring stratified by sex. **(A)** Serum fructose concentrations in male offspring. **(B)** UA concentrations in male offspring. **(C)** Leptin concentrations in male offspring. **(D)** Serum PINP concentration in male offspring. **(E)** Serum CTX concentration in male offspring. **(F)** Serum fructose concentrations in female offspring. **(G)** UA concentrations in female offspring. **(H)** Leptin concentrations in female offspring. **(I)** Serum PINP concentration in female offspring. **(J)** Serum CTX concentration in female offspring. Values are the means ± SDs. *n* = 8 per group. ^a^*P* < 0.05 (HFRU, LFRU, and GLU vs. CON). ^b^*P* < 0.05 (HFRU and LFRU vs. GLU). ^c^*P* < 0.05 (HFRU vs. LFRU).

There was no difference in serum fructose concentrations in both male and female offspring (Figures 4A, F). In both male and female offspring, the UA level was higher in the HFRU group than in the CON and GLU groups, and the UA level in the LFRU group was higher than in the CON groups (Figures 4B, G).

### 3.5. Leptin concentrations in dams and offspring

Compared with the CON and LFRU groups, the serum leptin of pregnant rats in the HFRU group was significantly increased (Figure 3C). The serum leptin of both male and female offspring of the HFRU and LFRU groups was significantly higher than those in the CON group, and the serum leptin of the offspring of the GLU group was significantly higher than those of the CON and LFRU groups (Figures 4C, H).

### 3.6. Circulating markers of bone metabolism in offspring

The PINP level was higher in male and female offspring in the HFRU and LFRU groups than in the CON group, and the PINP level was higher in male offspring in the LFRU group than in the GLU group (Figures 4D, I). The CTX level was higher in male and female offspring in the HFRU and LFRU groups than in the CON and GLU groups, and the CTX level of female offspring rats in the GLU group was significantly higher than that in the CON group (Figures 4E, J).

### 3.7. Bone histomorphology in offspring

#### 3.7.1. HE staining

**Figure 5:**
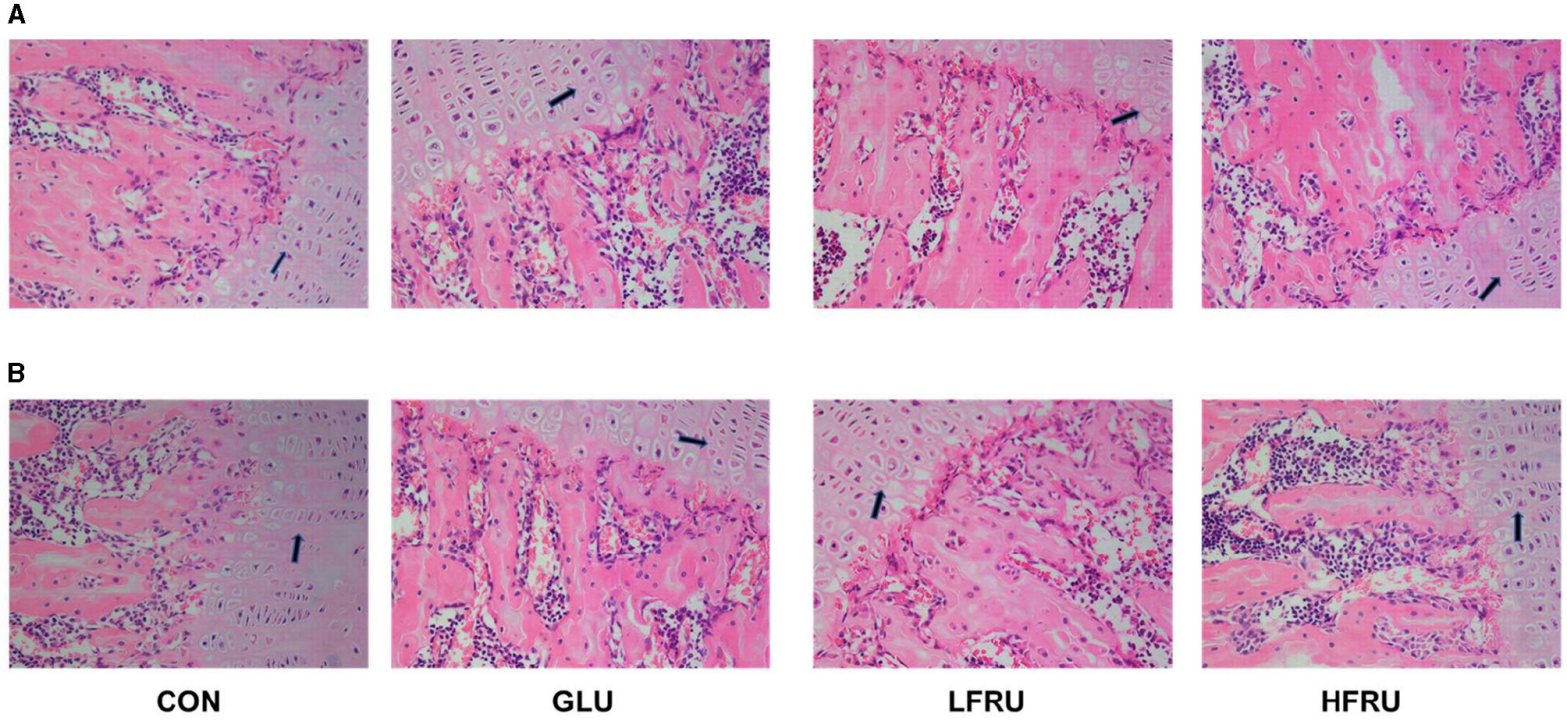
Effects of high-fructose consumption on cartilage. HE staining of the rats, scale bar, 400 μm. **(A)** Male offspring. **(B)** Female offspring. The arrow points to the chondrocyte proliferative zone.

HE staining showed that the growth plate of the metaphysis was abnormal and that the chondrocyte proliferative zone was reduced in the HFRU (Figure 5). We observed changes in both female and male rats.

#### 3.7.2. Trap staining

**Figure 6:**
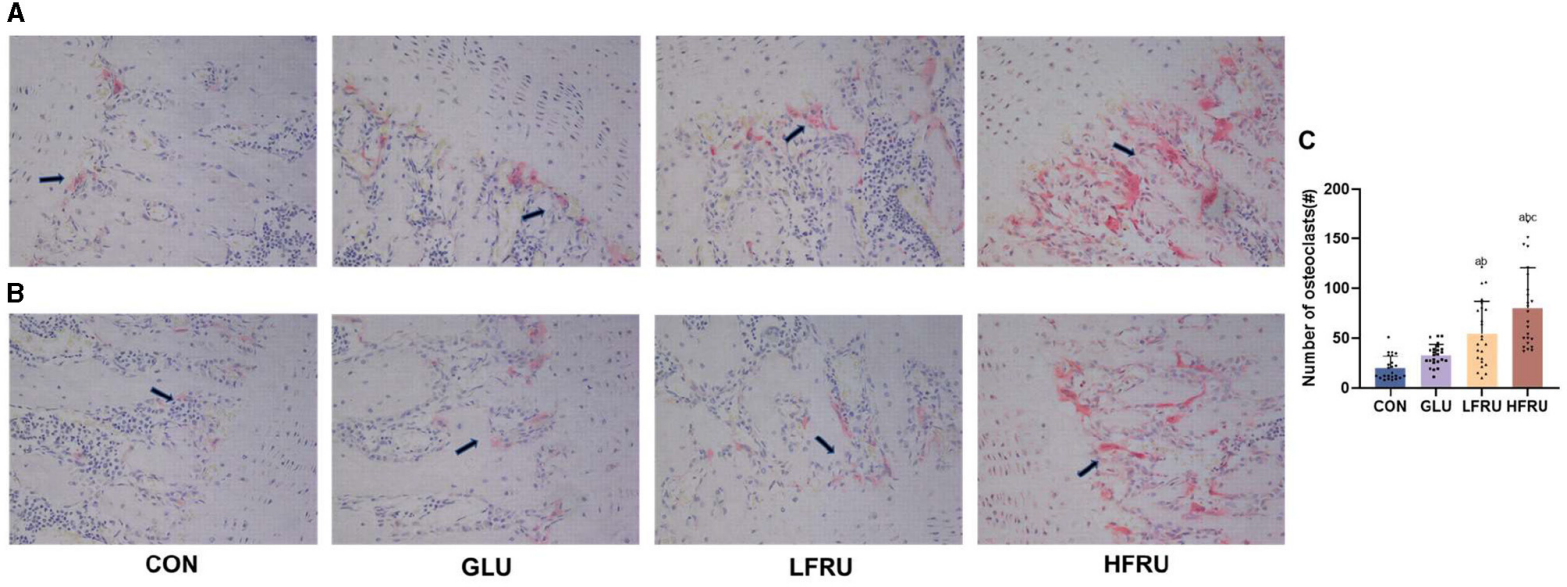
Effects of high-fructose consumption on osteoclasts. TRAP staining of rat tissues, scale bar, 400 μm. **(A)** Male offspring. **(B)** Female offspring. **(C)** Number of osteoclasts. The arrow points to the osteoclasts. Values are the means ± SDs. ^a^*P* < 0.05 (HFRU, LFRU, and GLU vs. CON). ^b^*P* < 0.05 (HFRU and LFRU vs. GLU). ^c^*P* < 0.05 (HFRU vs. LFRU).

In the HFRU group, many giant and multinucleated osteoclasts that were closely distributed were observed, and the osteoclasts had irregular shapes. Osteoclasts in the HFRU group showed an increase in the number of multinucleated cells, which indicated that the number of osteoclasts in the bone tissue had changed significantly (Figure 6).

### 3.8. Microstructural properties and volumetric bone mineral density in offspring by micro-CT

#### 3.8.1. Cortical bone compartment

**Figure 7:**
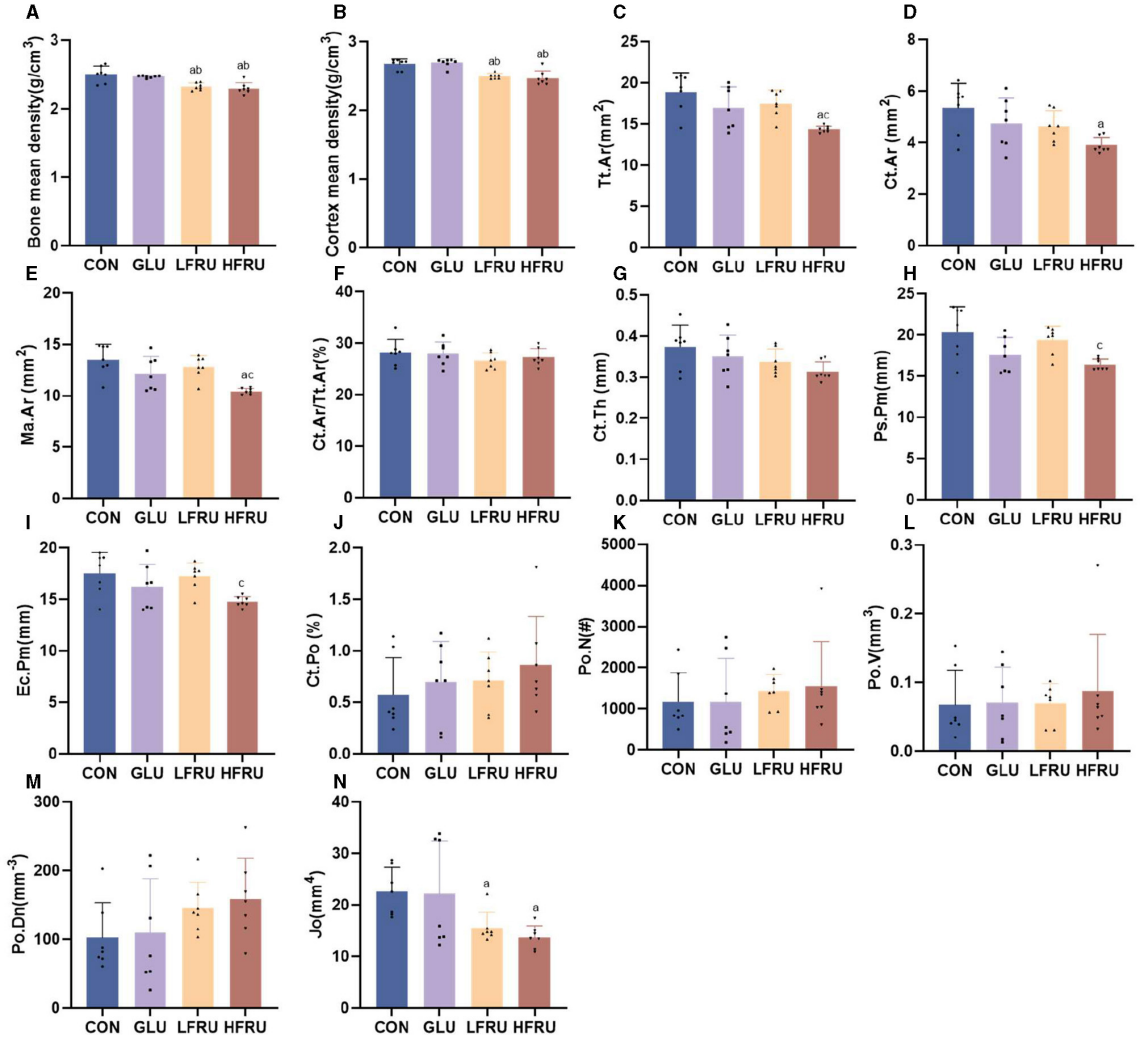
Evaluation of the microstructural properties of the male offspring cortical bone region (rat distal femur) by microcomputed tomography. **(A)** Bone mean density. **(B)** Cortex mean density. **(C)** Total cross-sectional area inside the periosteal envelope. **(D)** Cortical bone area. **(E)** Medullary (or marrow) area. **(F)** Cortical area fraction. **(G)** Average cortical thickness. **(H)** Periosteal perimeter. **(I)** Endocortical perimeter. **(J)** Cortical porosity Po.V/CT.V. **(K)** Pore number. **(L)** Total pore volume. **(M)** Pore density Po.N/CT.V. **(N)** Polar moment inertia. Values are the means ± SDs. *n* = 7 per group. ^a^*P* < 0.05 (HFRU, LFRU, and GLU vs. CON). ^b^*P* < 0.05 (HFRU and LFRU vs. GLU). ^c^*P* < 0.05 (HFRU vs. LFRU).

**Figure 8:**
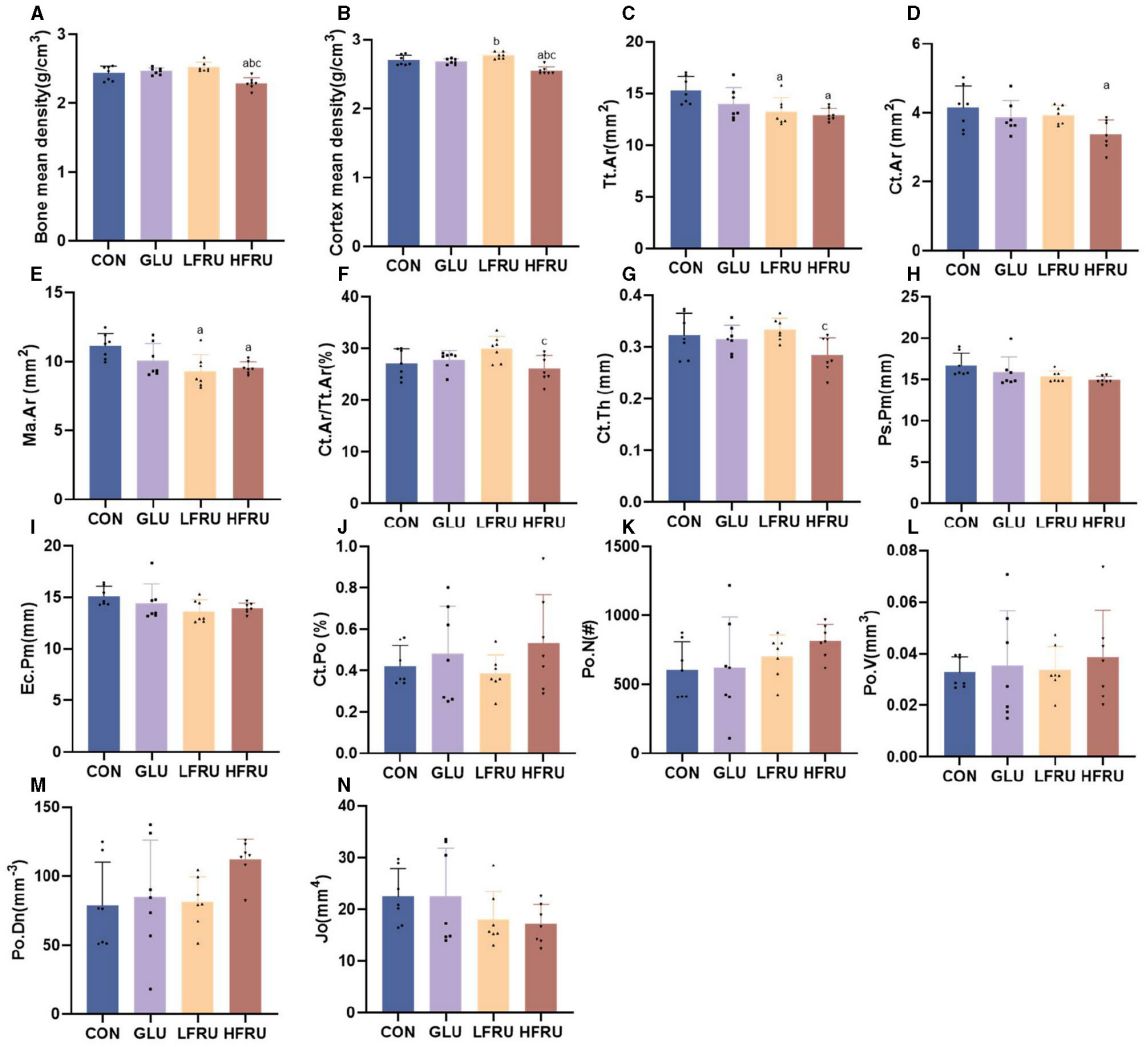
Evaluation of the microstructural properties of the female offspring cortical bone region (rat distal femur) by microcomputed tomography. **(A)** Bone mean density. **(B)** Cortex mean density. **(C)** Total cross-sectional area inside the periosteal envelope. **(D)** Cortical bone area. **(E)** Medullary (or marrow) area. **(F)** Cortical area fraction. **(G)** Average cortical thickness. **(H)** Periosteal perimeter. **(I)** Endocortical perimeter. **(J)** Cortical porosity Po.V/CT.V. **(K)** Pore number. **(L)** Total pore volume. **(M)** Pore density Po.N/CT.V. **(N)** Polar moment inertia. Values are the means ± SDs. *n* = 7 per group. ^a^*P* < 0.05 (HFRU, LFRU, and GLU vs. CON). ^b^*P* < 0.05 (HFRU and LFRU vs. GLU). ^c^*P* < 0.05 (HFRU vs. LFRU).

**Figure 9:**
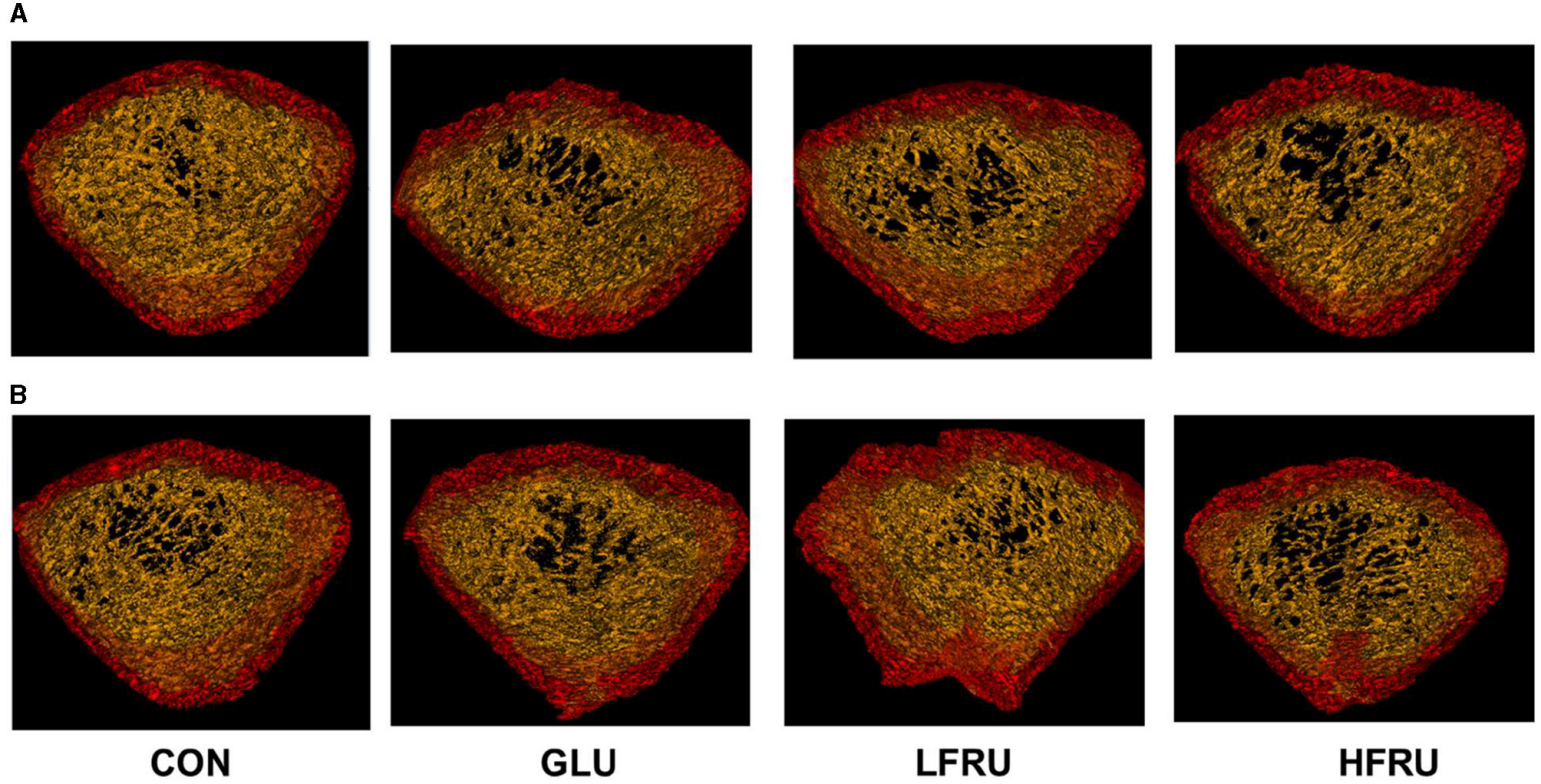
Micro-CT scanning images of the cortical bone compartment. **(A)** Male offspring. **(B)** Female offspring.

High fructose intake resulted in significant decreases in the measured microstructural properties of bone mineral density. The bone mean density and cortex mean density in the male offspring of the HFRU and LFRU groups were significantly lower than those in the CON and GLU groups (Figure 7A). The bone mean density and cortex mean density in the female offspring of the HFRU group were significantly lower than those in the CON, GLU, and LFRU groups (Figure 8A). In addition, it was also observed that the Tt.Ar, Ct.Ar, and Ma.Ar of both male and female offspring rats in the HFRU group were significantly lower than those in the CON group (Figures 7C–E, 8C–E). The Jo of male offspring rats in the HFRU group was significantly lower than that in the CON group (Figure 7N), but no difference was observed between the groups of female offspring rats in the HFRU group (Figure 8N). There was no significant difference in Ct.Ar/Ma.Ar, Ct.Po, Po.N, Po.V, or Po.Dn among the male and female offspring groups (Figures 7, 8). Figure 9 shows the micro-CT scanning images of the cortical bone compartment. The cortical bone area was lower in the LFRU group than in the CON group.

#### 3.8.2. Cancellous bone compartment

**Figure 10:**
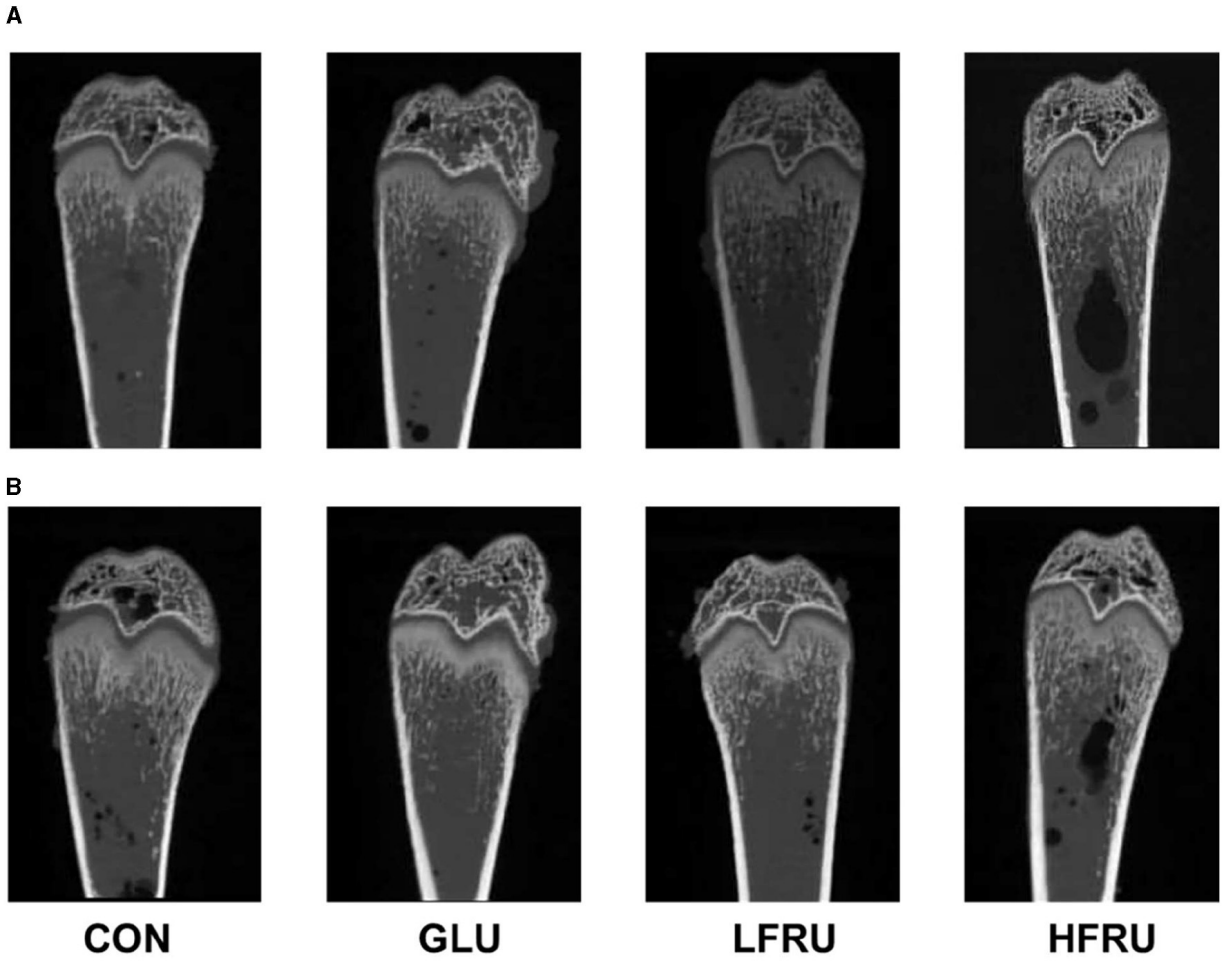
Micro-CT scanning images of the cancellous bone compartment. **(A)** Male offspring. **(B)** Female offspring.

**Figure 11:**
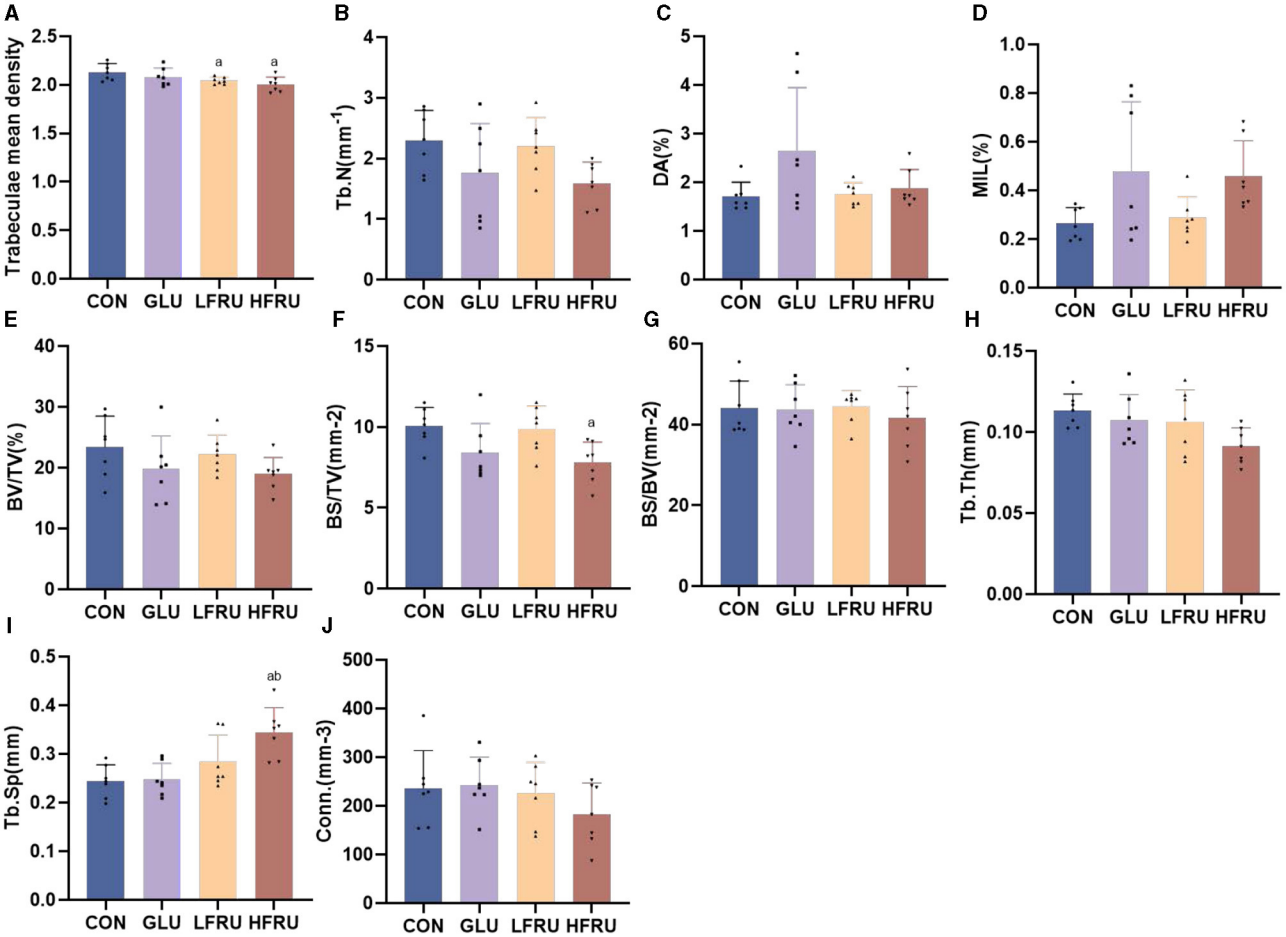
Evaluation of the microstructural properties of the male offspring cancellous bone region (rat distal femur) by microcomputed tomography. **(A)** Trabecular mean density. **(B)** Trabecular number. **(C)** Degree of anisotropy. **(D)** Mean intercept length. **(E)** Bone volumetric fraction. **(F)** Bone surface density. **(G)** Specific bone surface. **(H)** Trabecular thickness. **(I)** Trabecular spacing. **(J)** Connectivity density. Values are the means ± SDs. *n* = 7 per group. ^a^*P* < 0.05 (HFRU, LFRU, and GLU vs. CON). ^b^*P* < 0.05 (HFRU and LFRU vs. GLU).

**Figure 12:**
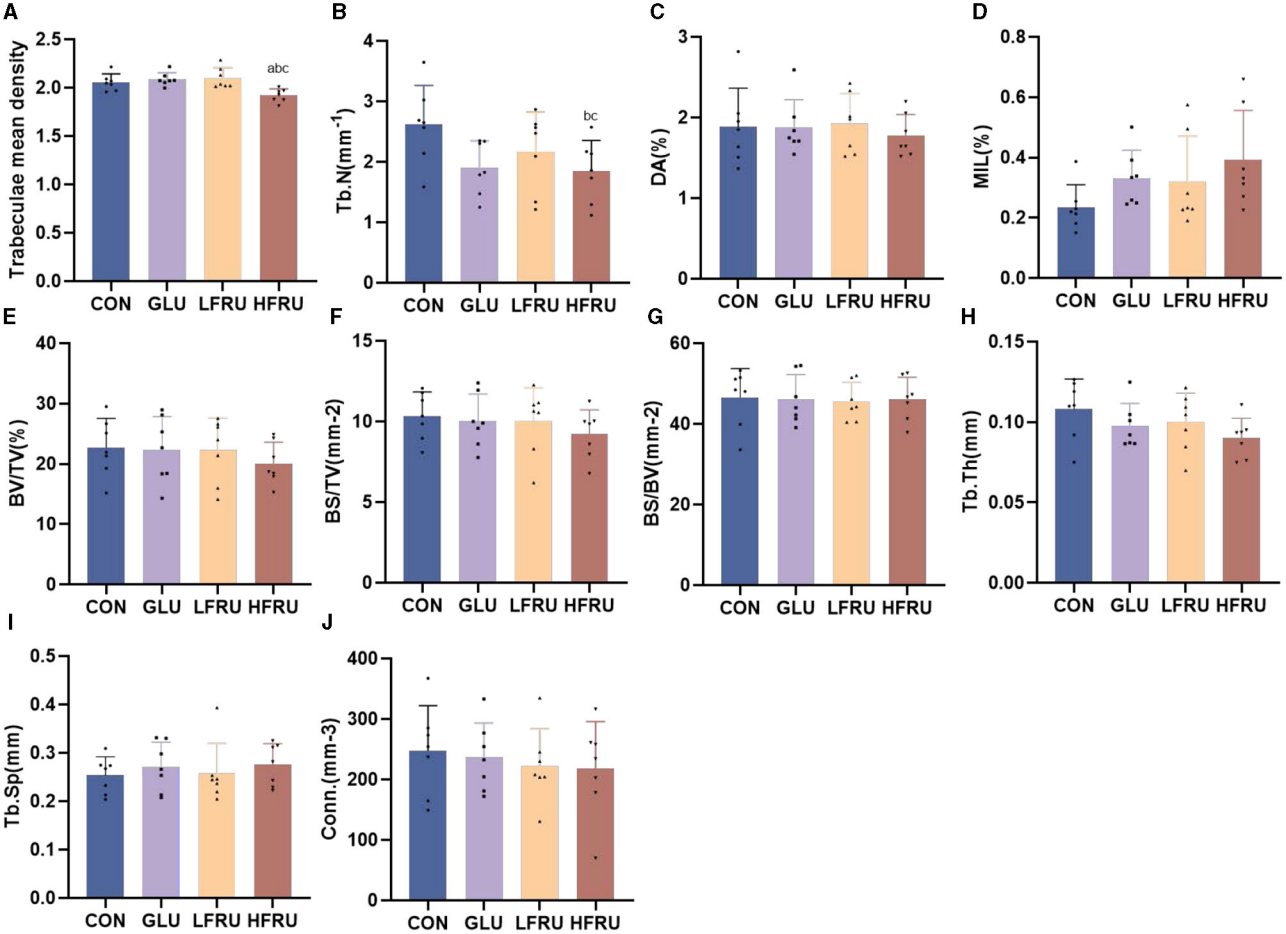
Evaluation of the microstructural properties of the female offspring cancellous bone region (rat distal femur) by microcomputed tomography. **(A)** Trabecular mean density. **(B)** Trabecular number. **(C)** Degree of anisotropy. **(D)** Mean intercept length. **(E)** Bone volumetric fraction. **(F)** Bone surface density. **(G)** Specific bone surface. **(H)** Trabecular thickness. **(I)** Trabecular spacing. **(J)** Connectivity density. Values are the means ± SDs. *n* = 7 per group. ^a^*P* < 0.05 (HFRU, LFRU, and GLU vs. CON). ^b^*P* < 0.05 (HFRU and LFRU vs. GLU). ^c^*P* < 0.05 (HFRU vs. LFRU).

The trabecular microarchitecture parameters of the offspring at 7 weeks of age are shown in Figure 10. There was no significant difference in the DA, MIL, BV/TV, BS/BV, Tb.Th, or Conn. in offspring among the male and female offspring groups (Figures 11, 12). The trabeculae mean density of male offspring rats in the HFRU and LFRU groups was significantly lower than that in the CON group (Figure 11A), while the trabeculae mean density of female offspring rats in the HFRU group was significantly lower than that in the CON, GLU, and LFRU groups (Figure 12A). The male offspring HFRU group had a lower BS/TV and a higher Tb.Sp than those in the CON group (Figures 11F, I), and the female offspring HFRU group had a lower Tb.N than those in the GLU and LFRU groups (Figure 12B).

## 4. Discussion

We investigated the effects of maternal high fructose intake on bone metabolism levels and cortical and trabecular bone properties in offspring rats. The results showed that maternal high fructose intake has lasting effects on the offspring's skeletal phenotype, with decreases in bone mean density, cortical mean density, trabecular mean density, Tt.Ar, Ct.Ar, and Ma.Ar, even when offspring are raised on a normal diet.

Fetal weight directly reflects embryonic development, and maternal undernutrition, gestational diabetes, and genetic factors may affect fetal birth weight (17–19). Although there was no significant difference in body weight among the four groups of rats during pregnancy, in our study, fetal weight was significantly lower in the HFRU group on day 21 of gestation, and offspring weight was significantly lower in the LFRU and HFRU groups on weeks 6 and 7. Although food intake was lower in the GLU, LFRU, and HFRU groups than in the CON group, energy intake during pregnancy was similar in the four groups when the energy of the gavage solution was added. According to previous studies, excessive or disproportionate dietary intake may affect placental ratios, impair placental supply capacity, and increase oxidative stress in the growing fetus. Thus, the intrauterine growth rate, birth weight, and survivability are reduced (20).

Bone microarchitecture is a predictor of bone quality and health, and micro-CT is a precise and non-destructive evaluation approach that can provide a comprehensive overview of the morphological and architectural characteristics of the bone (21). In our research, we found that cortical mean density, Tt.Ar, Ct.Ar, and Ma.Ar decreased in the cortical bone of the offspring rats in the HFRU group, which indicated that high fructose intake during pregnancy negatively affected cortical bone in the offspring, with effects that persisted into adolescence. Cortical structures are first formed by embryonic and maternal-specific influences during embryogenesis. During growth, the cortical composition changes as it merges, expands, and takes shape in the epiphyseal region (22). Excessive fructose intake during pregnancy can alter the microenvironment of embryonic development, thus affecting bone endochondral osteogenesis and cortical structure. As epiphyseal plate chondrocytes continue to divide, proliferate, and degenerate, osteoclasts, and osteoblasts continue to break down and absorb the calcified cartilage matrix from the lateral side of the bone marrow cavity, eventually forming bone trabeculae. The present results revealed a decreased trabecular BS in the HFRU group, which indicated an accelerated trabecular bone turnover. The trabecular microstructure assay in this study also showed significant decreases in trabecular mean density in the offspring of the HFRU group compared to the CON group. Our results demonstrated that high fructose intake during pregnancy not only negatively affected cortical bone but also impaired metaphyseal trabecular microstructure.

We further conducted histopathological exams in the offspring to investigate whether offspring endochondral osteogenesis was affected by maternal high fructose intake. HE staining was performed on the femur metaphysis. Osteogenesis of long bones begins during embryogenesis by endochondral ossification (23). The epiphyseal growth plate is the key structure for endochondral osteogenesis, which is mainly responsible for long bone growth (24). The length of the chondrocyte proliferative zone is an important factor in determining the length of the long bones (25). In our study, HE staining showed abnormal epiphyseal growth plates and reduced chondrocyte proliferation bands in the HFRU group. In the second trimester, the long bones undergo rapid cell division; this is called the “critical period.” There is a theory that bones are particularly susceptible to adverse environmental factors, such as changes in nutrient availability, during this period (26). Our results suggested that a maternal high-fructose diet can affect the proliferation of chondrocytes, thus affecting the offspring's bone development.

Bone mass can be permanently affected by nutritional changes during *in utero* development. By examining bone remodeling in offspring, we investigated how high fructose consumption in mothers affects fetal bone health. Bone remodeling is a lifelong process during which mature bone tissue is absorbed and new bone tissue is synthesized. In mammals, the ongoing process of bone remodeling begins with the appearance of osteoclasts (27). A decrease in bone mass is caused by decreased bone formation due to osteoblasts and increased bone resorption due to osteoclasts (28). The statistical analysis of osteoclast Trap staining showed that high-fructose intervention during pregnancy could increase the number of osteoclasts and the absorption area, which suggested that the bone loss caused by high fructose may be related to the enhancement of osteoclast function. Devlin et al. proposed that the diet of the mother impacts the skeletal phenotype of the offspring, potentially through “developmental programming” (29). Previous research also showed that perinatal programming could alter postnatal bone mass directly by increasing or decreasing bone cell numbers, proliferation, or growth (30, 31).

These findings were supported by further analysis of serum markers of bone turnover, including P1NP and CTX. CTX reflects osteoclastic bone resorption activity, and its elevation is consistent with increased osteoclastic activity and is an important biochemical marker of bone resorption. The amount of PINP in serum reflects the ability of osteoblasts to synthesize collagen and can be used to monitor osteoblast viability and bone formation (32, 33). In our study, although both PINP and CTX levels were elevated, the trend toward elevated CTX was more pronounced. We believed that maternal fructose intake can disrupt the balance between bone formation and bone resorption in offspring and thus have a greater effect on bone resorption than on bone production, resulting in bone loss.

It is challenging to interpret our data given that few previous animal studies have tested how a maternal high-fructose diet affects offspring bone mass. Previous studies have mainly focused on the effect of a maternal high-fat diet or protein restriction on the bone development of offspring. There is evidence that factors during the prenatal period, such as maternal nutritional status, may influence the bone health of offspring in direct and indirect ways (34). Research showed that the female offspring of mothers on HF diets had lower bone mineral content, but both male and female offspring had greater trabecular bone volume fractions (29). Mangu et al. found that compared with the control group, offspring in the maternal high-cholesterol diet group showed a reduction in bone mass and bone quality at all ages (20). This is the first research to show that the cross-generational effect of offspring skeletal development is secondary to maternal high fructose intake.

According to the “fetal origins” hypothesis, changes in fetal nutrition and endocrine status can lead to developmental adaptations, making offspring more likely to develop cardiovascular, metabolic, and endocrine diseases later in life (35). The mechanism underlying this effect is called “fetal programming” (36). The effect of high-fructose exposure during pregnancy on bone development in offspring rats may result from the pathogenic gene programming of bone formation and resorption. Therefore, it is important to answer whether the skeletal phenotype of HFRU offspring results primarily from the perinatal developmental programming of bone or whether we are observing secondary effects of perinatal programming of metabolic pathways, which then influence skeletal metabolism.

We suppose that the offspring's bone damage is caused by changes in metabolic and endogenic alterations. It has been reported that excess maternal fructose consumption can result in disordered glucose metabolism in offspring (37). In our study, fasting glucose, fasting insulin, and HOMA-IR were significantly increased in the HFRU group of offspring rats, consistent with a previous study (38). These results supported the idea that a maternal diet high in fructose can cause insulin resistance in offspring. Insulin signaling regulates bone formation by osteoblasts and bone resorption by osteoclasts. Insulin receptors on the osteoblast surface are required for osteoblast proliferation, survival, and differentiation (39). A mouse model showed that hyperinsulinemia and insulin resistance caused reduced bone turnover and led to increased bone fragility by increasing cortical porosity or other defects in bone microarchitecture (40). A longitudinal study showed that insulin resistance may be detrimental to bone development through puberty in boys (41). Thus, insulin resistance in offspring caused by high fructose during pregnancy may be one of the mechanisms that inhibit bone growth and development in offspring.

Furthermore, our study found that high fructose intake during pregnancy may induce leptin resistance in both dams and offspring, resulting in impaired leptin signaling. Our results were consistent with previous research (42). Leptin regulates appetite, weight, body metabolism, and reproductive function. As a key regulator of energy intake, leptin also has a multifaceted impact on bone metabolism, regulating bone remodeling and bone mass in rodents and humans. Leptin can inhibit bone formation by activating the sympathetic nervous system (SNS) while stimulating bone resorption through skeletal adrenergic receptors on osteoblasts and indirectly regulating bone metabolism through the hypothalamus (43). Elevated leptin levels may also be one of the causes of bone development in offspring. However, the exact mechanism requires further study.

In addition to affecting glucose metabolism, another mechanism by which a high-fructose diet disturbs the fetal programming of bone may be an increase in UA in offspring. Our study found that offspring rats in the LFRU and HFRU groups had varying degrees of UA elevation compared with those in the CON group. In contrast to other sugars, the metabolism of fructose stimulates UA production (44). The increase in UA concentration in offspring may be due to (a) the metabolism of higher circulating free fatty acids, which is caused by the increased intracellular ATP resulting from excessive fructose, or (b) UA being transferred from the mother to the fetus through the placenta during pregnancy (45). Normal levels of UA in the body may be an osteoprotective factor (46). However, as a pro-oxidant factor, UA can induce oxidative stress inside cells and cause tissue damage (47). It has also been found that high UA decreases the levels of bone formation markers and decreases the bone conversion rate (48). The results of elevated UA in our study are consistent with a trend toward diminished bone microarchitecture, suggesting that high fructose intake may affect bone homeostasis by increasing UA levels.

In the study of Zhang et al., in rats fed a high-fructose diet during pregnancy, UA concentrations were markedly increased in the placenta and fetal serum, and this was associated with a significant elevation in the concentration of the lipid peroxidation product (MDA) and decreased activities of the antioxidant enzymes (SOD, CAT, and GSH-Px) (49). Bone development disorders in the embryo are believed to be mediated by oxidative stress damage. Increasing numbers of studies have reported that fetal bone is extremely sensitive to environmental influences during pregnancy, such that adverse exposures such as oxidative stress will increase the risk of skeletal disorders. The alteration of the redox state causes systemic changes that can coordinate osteoblast differentiation or osteoclast activity related to the bone remodeling process (50, 51). We propose that a maternal high-fructose diet can impair cartilage proliferation and bone homeostasis by increasing fetal oxidative stress and then affecting offspring bone mass and microstructure. However, the underlying molecular and biological mechanisms need further study.

A final possibility is that a maternal high-fructose diet might alter the offspring's bone mass through epigenetic mechanisms. Although much more research is needed, there is some evidence that DNA methylation and other epigenetic mechanisms can induce lifelong changes in the transcription of genes influencing bone mass. The epigenetic effects of a maternal high-fructose diet on bone development in offspring require further study.

Our study shows that when stratified by sex, the bone changes have less difference between male and female offspring. A trend in the effect of fructose on mean bone density, mean cortical density, and trabecular bone density was observed. However, in female rats, there was a slight trend of density enhancement in the LFRU group compared to the CON group, while the HFRU group remained suppressed. This difference may be due to the different sensitivities of male and female rats to fructose. Few studies have investigated whether fructose ingestion has a sex-specific effect on metabolism. Silvia Rodrigo et al. observed that male offspring from mothers who consumed fructose had elevated plasma HDL cholesterol levels, whereas female offspring from mothers who consumed fructose had lower non-HDL cholesterol levels (52). There is evidence that male and female offspring respond differently to the same stimuli in early life. However, it is difficult to determine whether high fructose intake leads to a true sex-specific response (53, 54).

Our results showed that a high-fructose diet during pregnancy had adverse effects on bone development and bone mass in offspring, which is a potential etiological basis for osteoporosis in adulthood. This offers guidance for a healthy diet during pregnancy. This study also has certain limitations; for example, the conception time of female rats was not completely consistent, but the time of offspring sacrifice was relatively constant, resulting in inconsistency in offspring age. In addition, because our study followed offspring for only 7 weeks, further long-term follow-up is needed. Finally, the molecular mechanism should be given more attention. In the future, we will focus on the level of inflammation and key pathways for bone development.

## 5. Conclusion

Our study concludes that intrauterine environment changes due to high fructose consumption have a negative impact on the bone metabolic microenvironment and trabecular microstructure, which continues into later life. Fruits, processed meals, and beverages that are high in fructose should be appropriately limited for pregnant women.

## Data availability statement

The original contributions presented in the study are included in the article/supplementary material, further inquiries can be directed to the corresponding author.

## Ethics statement

The animal study was reviewed and approved by Animal Experimentation Ethics Committee of the Medical College of Qingdao University.

## Author contributions

YL and LH conceived and planned the experiments. XL processed and analyzed the data. XL, YC, and CL took the lead in writing the manuscript. TG, XJ, ZZ, QS, and LH provided critical feedback and helped shape the research, analysis, and manuscript. The results of this study have been presented clearly, honestly, and without fabrication, falsification, or inappropriate data manipulation. All authors have read and agreed to the published version of the manuscript.

## References

[1] Patterson ME Yee JK Wahjudi P Mao CS Lee WP. Acute metabolic responses to high fructose corn syrup ingestion in adolescents with overweight/obesity and diabetes. J Nutr Intermed Metab. (2018) 14:1–7. 10.1016/j.jnim.2018.08.004 31058204 PMC6497393

[2] Huang WQ Lu Y Xu M Huang J Su YX Zhang CX. Excessive fruit consumption during the second trimester is associated with increased likelihood of gestational diabetes mellitus: a prospective study. Sci Rep. (2017) 7:43620. 10.1038/srep43620 28272552 PMC5341573

[3] Liu X Yue J Wang H. Development pattern and regional characteristics of global high fructose sweeteners production. Sugar Crops China. (2021) 43:76–81. 10.13570/j.cnki.scc.2021.02.013

[4] Sun H Chen S Pang X Dong H Cai C Bai D. Association between fruit intake during pregnancy and blood glucose metabolism. Wei Sheng Yan Jiu. (2022) 51:550–5. 10.19813/j.cnki.weishengyanjiu.2022.04.009 36047257

[5] Zhou X Chen R Zhong C Wu J Li X Li Q. Fresh fruit intake in pregnancy and association with gestational diabetes mellitus: a prospective cohort study. Nutrition. (2019) 60:129–35. 10.1016/j.nut.2018.09.022 30572275

[6] Gao T Tian C Tian G Ma L Xu L Liu W. Excessive fructose intake inhibits skeletal development in adolescent rats via gut microbiota and energy metabolism. Front Microbiol. (2022) 13:952892. 10.3389/fmicb.2022.952892 36187951 PMC9519145

[7] Han X Feng Z Chen Y Zhu L Li X Wang X. Effects of high-fructose corn syrup on bone health and gastrointestinal microbiota in growing male mice. Front Nutr. (2022) 9:829396. 10.3389/fnut.2022.829396 35433775 PMC9005738

[8] TianLWangCXieYWanSZhangKYuX. High fructose and high fat exert different effects on changes in trabecular bone micro-structure. J Nutr Health Aging. (2018) 22:361–70. 10.1007/s12603-017-0933-029484349

[9] Prentice A Schoenmakers I Ann Laskey M De Bono S Ginty F Goldberg GR. Symposium On ‘nutrition and health in children and adolescents' session 1: nutrition in growth and development nutrition and bone growth and development. Proc Nutr Soc. (2007) 65:348–60. 10.1079/PNS2006519 PMC2039894 17181901

[10] Barker DJ. The origins of the developmental origins theory. J Intern Med. (2007) 261:412–7. 10.1111/j.1365-2796.2007.01809.x 17444880

[11] Chen JR Zhang J Lazarenko OP Kang P Blackburn ML Ronis MJ. Inhibition of fetal bone development through epigenetic down-regulation of hoxa10 in obese rats fed high-fat diet. FASEB J. (2012) 26:1131–41. 10.1096/fj.11-197822 22131269

[12] Chen JR Lazarenko OP Zhao H Alund AW Shankar K. Maternal obesity impairs skeletal development in adult offspring. J Endocrinol. (2018) 239:33–47. 10.1530/JOE-18-0244 30307152 PMC6145139

[13] Lineker C Kerr PM Nguyen P Bloor I Astbury S Patel N. High fructose consumption in pregnancy alters the perinatal environment without increasing metabolic disease in the offspring. Reprod Fertil Dev. (2016) 28:2007–15. 10.1071/RD15119 26143929

[14] Tappy L Lê KA. Metabolic effects of fructose and the worldwide increase in obesity. Physiol Rev. (2010) 90:23–46. 10.1152/physrev.00019.2009 20086073

[15] Herman MA Birnbaum MJ. Molecular aspects of fructose metabolism and metabolic disease. Cell Metab. (2021) 33:2329–54. 10.1016/j.cmet.2021.09.010 34619074 PMC8665132

[16] Faeh D Minehira K Schwarz JM Periasamy R Park S Tappy L. Effect of fructose overfeeding and fish oil administration on hepatic de novo lipogenesis and insulin sensitivity in healthy men. Diabetes. (2005) 54:1907–13. 10.2337/diabetes.54.7.1907 15983189

[17] Warrington NM Beaumont RN Horikoshi M Day FR Helgeland Ø Laurin C. Maternal and fetal genetic effects on birth weight and their relevance to cardio-metabolic risk factors. Nat Genet. (2019) 51:804–14. 10.1038/s41588-019-0403-1 31043758 PMC6522365

[18] Roland MC Friis CM Godang K Bollerslev J Haugen G Henriksen T. Maternal factors associated with fetal growth and birthweight are independent determinants of placental weight and exhibit differential effects by fetal sex. PLoS ONE. (2014) 9:E87303. 10.1371/journal.pone.0087303 24516548 PMC3916298

[19] Workalemahu T Grantz KL Grewal J Zhang C Louis GMB Tekola-Ayele F. Genetic and environmental influences on fetal growth vary during sensitive periods in pregnancy. Sci Rep. (2018) 8:7274. 10.1038/s41598-018-25706-z 29740100 PMC5940684

[20] Mangu SR Patel K Sukhdeo SV Savitha MR Sharan K. Maternal high-cholesterol diet negatively programs offspring bone development and downregulates hedgehog signaling in osteoblasts. J Biol Chem. (2022) 298:102324. 10.1016/j.jbc.2022.102324 35931113 PMC9440389

[21] Chen C Kim WK. The application of micro-ct in egg-laying hen bone analysis: introducing an automated bone separation algorithm. Poult Sci. (2020) 99:5175–83. 10.1016/j.psj.2020.08.047 33142433 PMC7647928

[22] Isojima T Sims NA. Cortical bone development, maintenance and porosity: genetic alterations in humans and mice influencing chondrocytes, osteoclasts, osteoblasts and osteocytes. Cell Mol Life Sci. (2021) 78:5755–73. 10.1007/s00018-021-03884-w 34196732 PMC11073036

[23] Provot S Schipani E. Molecular mechanisms of endochondral bone development. Biochem Biophys Res Commun. (2005) 328:658–65. 10.1016/j.bbrc.2004.11.068 15694399

[24] Rauch F. Bone growth in length and width: the yin and yang of bone stability. J Musculoskelet Neuronal Interact. (2005) 5:194–201. 16172510

[25] Nilsson O Baron J. Fundamental limits on longitudinal bone growth: growth plate senescence and epiphyseal fusion. Trends Endocrinol Metab. (2004) 15:370–4. 10.1016/j.tem.2004.08.004 15380808

[26] Goodfellow LR Earl S Cooper C Harvey NC. Maternal diet, behaviour and offspring skeletal health. Int J Environ Res Public Health. (2010) 7:1760–72. 10.3390/ijerph7041760 20617058 PMC2872349

[27] Salhotra A Shah HN Levi B Longaker MT. Mechanisms of bone development and repair. Nat Rev Mol Cell Biol. (2020) 21:696–711. 10.1038/s41580-020-00279-w 32901139 PMC7699981

[28] Xiao W Wang Y Pacios S Li S Graves DT. Cellular and molecular aspects of bone remodeling. Front Oral Biol. (2016) 18:9–16. 10.1159/000351895 26599113 PMC10754210

[29] Devlin MJ Grasemann C Cloutier AM Louis L Alm C Palmert MR. Maternal perinatal diet induces developmental programming of bone architecture. J Endocrinol. (2013) 217:69–81. 10.1530/JOE-12-0403 23503967 PMC3792707

[30] Sayer AA Cooper C. Fetal programming of body composition and musculoskeletal development. Early Hum Dev. (2005) 81:735–44. 10.1016/j.earlhumdev.2005.07.003 16081228

[31] Javaid MK Cooper C. Prenatal and childhood influences on osteoporosis. Best Pract Res Clin Endocrinol Metab. (2002) 16:349–67. 10.1053/beem.2002.0199 12064897

[32] Hanusch B Prediger M Tuck SP Walker J Mcnally R Datta HK. Bone turnover markers as determinants of bone density and fracture in men with distal forearm fractures: the pathogenesis examined in the mr f study. Osteoporos Int. (2021) 32:2267–77. 10.1007/s00198-021-06001-6 33990874

[33] Hojsager FD Rand MS Pedersen SB Nissen N Jorgensen NR. Fracture-induced changes in biomarkers ctx, pinp, oc, and bap-a systematic review. Osteoporos Int. (2019) 30:2381–9. 10.1007/s00198-019-05132-1 31446441

[34] Masztalerz-Kozubek D Zielinska-Pukos MA Hamulka J. Maternal diet, nutritional status, and birth-related factors influencing offspring's bone mineral density: a narrative review of observational, cohort, and randomized controlled trials. Nutrients. (2021) 13:2302. 10.3390/nu13072302 34371812 PMC8308284

[35] Shook LL Kislal S Edlow AG. Fetal brain and placental programming in maternal obesity: a review of human and animal model studies. Prenat Diagn. (2020) 40:1126–37. 10.1002/pd.5724 32362000 PMC7606714

[36] Monteiro LJ Norman JE Rice GE Illanes SE. Fetal programming and gestational diabetes mellitus. Placenta. (2016) 48:S54–60. 10.1016/j.placenta.2015.11.015 26724985

[37] Saad AF Dickerson J Kechichian TB Yin H Gamble P Salazar A. High-fructose diet in pregnancy leads to fetal programming of hypertension, insulin resistance, and obesity in adult offspring. Am J Obstet Gynecol. (2016) 215:378 E1–6. 10.1016/j.ajog.2016.03.038 27060421

[38] Softic S Stanhope KL Boucher J Divanovic S Lanaspa MA Johnson RJ. Fructose and hepatic insulin resistance. Crit Rev Clin Lab Sci. (2020) 57:308–22. 10.1080/10408363.2019.1711360 31935149 PMC7774304

[39] Jones RH Ozanne SE. Fetal programming of glucose-insulin metabolism. Mol Cell Endocrinol. (2009) 297:4–9. 10.1016/j.mce.2008.06.020 18662742

[40] Huang S Kaw M Harris MT Ebraheim N Mcinerney MF Najjar SM. Decreased osteoclastogenesis and high bone mass in mice with impaired insulin clearance due to liver-specific inactivation to ceacam1. Bone. (2010) 46:1138–45. 10.1016/j.bone.2009.12.020 20044046 PMC2862391

[41] Ronne MS Heidemann M Lylloff L Schou AJ Tarp J Bugge A. Bone mass development is sensitive to insulin resistance in adolescent boys. Bone. (2019) 122:1–7. 10.1016/j.bone.2019.02.005 30738213

[42] Rodríguez L Otero P Panadero MI Rodrigo S Álvarez-Millán JJ Bocos C. Maternal fructose intake induces insulin resistance and oxidative stress in male, but not female, offspring. J Nutr Metab. (2015) 2015:158091. 10.1155/2015/158091 25763281 PMC4339788

[43] Ducy P Amling M Takeda S Priemel M Schilling AF Beil FT. Leptin inhibits bone formation through a hypothalamic relay: a central control of bone mass. Cell. (2000) 100:197–207. 10.1016/S0092-8674(00)81558-5 10660043

[44] Lecoultre V Egli L Theytaz F Despland C Schneiter P Tappy L. Fructose-induced hyperuricemia is associated with a decreased renal uric acid excretion in humans. Diabetes Care. (2013) 36:E149–50. 10.2337/dc13-0866 23970726 PMC3747900

[45] Smith EVL Dyson RM Berry MJ Gray C. Fructose consumption during pregnancy influences milk lipid composition and offspring lipid profiles in guinea pigs. Front Endocrinol. (2020) 11:550. 10.3389/fendo.2020.00550 32849314 PMC7431635

[46] Lin KM Lu CL Hung KC Wu PC Pan CF Wu CJ. The paradoxical role of uric acid in osteoporosis. Nutrients. (2019) 11. 10.3390/nu11092111 31491937 PMC6769742

[47] Johnson RJ Perez-Pozo SE Sautin YY Manitius J Sanchez-Lozada LG Feig DI. Hypothesis: could excessive fructose intake and uric acid cause type 2 diabetes? Endocr Rev. (2009) 30:96–116. 10.1210/er.2008-0033 19151107 PMC2647706

[48] Veronese N Carraro S Bano G Trevisan C Solmi M Luchini C. Hyperuricemia protects against low bone mineral density, osteoporosis and fractures: a systematic review and meta-analysis. Eur J Clin Invest. (2016) 46:920–30. 10.1111/eci.12677 27636234

[49] Liu S Zhang H Yan B Zhao H Wang Y Gao T. Maternal high-fructose consumption provokes placental oxidative stress resulting in asymmetrical fetal growth restriction in rats. J Clin Biochem Nutr. (2021) 69:68–76. 10.3164/jcbn.21-19 34376916 PMC8325765

[50] Sheppard AJ Barfield AM Barton S Dong Y. Understanding reactive oxygen species in bone regeneration: a glance at potential therapeutics and bioengineering applications. Front Bioeng Biotechnol. (2022) 10:836764. 10.3389/fbioe.2022.836764 35198545 PMC8859442

[51] Schreurs AS Torres S Truong T Moyer EL Kumar A Tahimic CGT. Skeletal tissue regulation by catalase overexpression in mitochondria. Am J Physiol Cell Physiol. (2020) 319:C734–45. 10.1152/ajpcell.00068.2020 32783660

[52] Rodrigo S Fauste E De La Cuesta M Rodriguez L Alvarez-Millan JJ Panadero MI. Maternal fructose induces gender-dependent changes in both lxralpha promoter methylation and cholesterol metabolism in progeny. J Nutr Biochem. (2018) 61:163–72. 10.1016/j.jnutbio.2018.08.011 30236873

[53] Pinnick KE Hodson L. Challenging metabolic tissues with fructose: tissue-specific and sex-specific responses. J Physiol. (2019) 597:3527–37. 10.1113/JP277115 30883738

[54] Vickers MH Clayton ZE Yap C Sloboda DM. Maternal fructose intake during pregnancy and lactation alters placental growth and leads to sex-specific changes in fetal and neonatal endocrine function. Endocrinology. (2011) 152:1378–87. 10.1210/en.2010-1093 21303952

